# Evaluation of compliance of CONSORT-CHM formula 2017 in randomized controlled trials of Chinese herbal medicine formulas: protocol of a five-year review

**DOI:** 10.3389/fphar.2024.1287262

**Published:** 2024-02-23

**Authors:** Juan Wang, Chung Wah Cheng, Yalin Jiao, Dongni Shi, Yaochen Wang, Han Li, Nana Wang, Xihong Wang, Yuqin Li, Feng Liang, Shufeng Luo, Fei Han, Ji Li, Ping Wang, Aiping Lyu, Zhaoxiang Bian, Xuan Zhang

**Affiliations:** ^1^ Chinese EQUATOR Centre, Hong Kong Baptist University, Hong Kong SAR, China; ^2^ School of Chinese Medicine, Beijing University of Chinese Medicine, Beijing, China; ^3^ School of Traditional Chinese Medicine, Tianjin University of Chinese Medicine, Beijing, China; ^4^ School of Chinese Medicine, Hong Kong Baptist University, Hong Kong SAR, China; ^5^ Department of Pediatrics, Guang’anmen Hospital, China Academy of Chinese Medical Sciences, Beijing, China; ^6^ Xiyuan Hospital, China Academy of Chinese Medical Sciences, Beijing, China; ^7^ Vincent V.C. Woo Chinese Medicine Clinical Research Institute, School of Chinese Medicine, Hong Kong Baptist University, Hong Kong SAR, China

**Keywords:** CONSORT-CHM formula 2017, Chinese herbal medicine formula (CHMF), reporting guideline, quality control, Chinese medicine

## Abstract

**Background:** The CONSORT Extension for Chinese Herbal Medicine Formula 2017 (CONSORT-CHM Formula 2017) has established a reporting standard for randomized controlled trials (RCTs) of Chinese Herbal Medicine Formula (CHMF) interventions; however, its adherence and implications for the design and execution of study design remain ambiguous. It is necessary to evaluate the level of compliance with the CONSORT-CHM Formula 2017 in RCTs conducted over the past 5 years, and to determine the reporting quality of clinical trials in this field.

**Methods:** First, a systematic search is conducted for RCTs on CHMF in EBM Reviews, Allied and Complementary Medicine (AMED), Embase, Ovid-MEDLINE(R), Wanfang data, China National Knowledge Infrastructure (CNKI), VIP Chinese Medical Journal Database (VIP) and Chinese Biomedical Literature (CBM) database, that encompassed CHMF interventional RCTs published from 1 January 2018 to 8 June 2022, with language restriction to English or Chinese. Second, a descriptive analysis will be performed regarding the study design and general characteristics of the included trials. Third, for the quality assessment, we have subdivided the CONSORT-CHM Formula 2017 checklist (consisting of 22 extended items) into a total of 42 sub-questions to facilitate scoring, with a specific focus on the description, quality control, and safety assessment of CHMF interventions. Professional training and a pilot test on 100 randomly selected articles will be provided for all reviewers. Throughout this process, a standard operating procedure (SOP) for quality assessment will be developed to ensure consistency. Each item will be assessed by two reviewers in a paired back-to-back manner, and the compliance rate will be calculated to assess inter-rater agreement.

**Discussion:** This review will identify the current reporting characteristics and quality of CHMF interventional studies and further evaluate the impact of CONSORT-CHM Formula 2017. The results may provide suggestions for future application or promotion of the guideline.

Registration: The study has been registered on Open Science Framework (https://osf.io/xpn7f).

## Introduction

Traditional Chinese medicine (TCM) is a distinctive and comprehensive medical system that holds a crucial role in every level of healthcare services (World Health Organization, 2013). Chinese Herbal Medicine Formulas (CHMF) represent the most common form of TCM intervention, which is specific groupings of Chinese medicinal substances based on pattern differentiation. However, due to the unique theories of TCM and the complexity of CHMF, conducting and reporting clinical trials on them has been challenging in the past (Wang et al., 2007; He et al., 2011). In response to this issue, a new CONSORT (Consolidated Standards of Reporting Trials) extension, namely “CONSORT Extension for Chinese Herbal Medicine Formulas 2017 (CONSORT-CHM Formula 2017)” was developed. This reporting guideline incorporates the principles of TCM pattern and the characteristics of CHMF, aiming to enhance proper reporting of CHMF interventional studies. The guideline includes a reporting checklist, a detailed explanation and elaboration document, and available published examples of good reporting in English, along with translations in simplified Chinese and traditional Chinese (Cheng et al., 2017a; Cheng et al., 2017a; Cheng et al., 2017b).

The CONSORT-CHM Formula 2017 has garnered considerable attention and sparked increasing discussion (Wu et al., 2017). Experts commented that this reporting guideline could improve the design and execution of clinical trials involving CHMF and contribute to the advancement of TCM (Zhang and Shang, 2017; Wang and Jin, 2019). Prof. Klaus Linde, from Charité University Medical Center in Germany, has praised that “*The CONSORT extension for CHM Formulas 2017 is a highly welcome, detailed, and well-structured proposal*” (Linde and Brinkhaus, 2017). Various authoritative bodies, whether at the national or international level, have recommended the CONSORT-CHM Formula 2017 as a guiding tool for the practice, reporting of trials, and quality assessment of randomized controlled trials (RCTs) involving CHMF interventions, such as the National Administration of Traditional Chinese Medicine, the China Association of Chinese Medicine, the World Health Organization (WHO), the Evidence-Based Medicine Centre of Traditional Chinese Medicine, and the Korea Institute of Oriental Medicine in South Korea (Zhi and Xie, 2020; Lee et al., 2021; Integrated Health Services, 2022; National Administration of Traditional Chinese Medicine, 2023).

However, recent systematic reviews on various conditions, including eczema, essential hypertension, stroke, acute upper respiratory infection in children, prostate cancer, and male infertility, have highlighted the disappointing reporting quality of RCTs involving CHMF (Li et al., 2020; Xu et al., 2020; Tian et al., 2022; Yu et al., 2022; Yan et al., 2023; Zhang et al., 2023). Specific flaws identified include: 1) inadequate reporting on study design (such as the method of randomization), which easily arise a risk of bias that could impact the reliability and validity of the findings (Li et al., 2020; Zhang et al., 2023). 2) a lack of awareness among authors regarding the detail description of CHMF interventions. Furthermore, some scholars tried to explore the impact of the release of CONSORT-CHM Formula 2017, yielding divergent conclusions. In a review of chronic atrophic gastritis, it was suggested that the release of CONSORT-CHM Formula 2017 did not have a significant impact on the overall report quality (OR = 1.07, 95% CI (0.98, 1.17), *p* = 0.121). However, a notable difference was observed in the reporting rate of items specifically related to CHMF (OR = 1.29, 95% CI (1.06, 1.58), *p* = 0.011). It is worth highlighting that, among these reviews, only one undertook an analysis of reporting quality both before and after the publication of the CONSORT-CHM Formula 2017, rendering it challenging to definitively ascertain the guideline’s influence (Zhao et al., 2023).

Hence, to gain a more comprehensive understanding of the present utilization and impact of CONSORT-CHM Formula 2017 on the reporting quality of RCTs involving CHMF interventions, a review focusing on the most recent 5 years becomes imperative. The objective of this review is to ascertain the extent to which CONSORT-CHM Formula 2017 items have been included in reports and to evaluate the level of reporting compliance since its issuance in 2017. The outcomes of this review are anticipated to provide valuable insights and recommendations for the broader application and future promotion of CONSORT-CHM Formula 2017.

## Methods

The proposed review will be conducted referred to the Joanna Briggs Institute (JBI) methodology framework (Arksey and O’Malley, 2005; Peters et al., 2015), while this protocol is drafted in line with the Preferred Reporting Items for Systematic Reviews and Meta-Analyses extension for Protocol (PRISMA-P) (Shamseer et al., 2015). The study has been registered on Open Science Framework (https://osf.io/xpn7f) and will be updated with amendments if required. The reporting of the final results of this review will be according to the Preferred Reporting Items for Systematic Reviews and Meta-Analyses extension for Scoping Reviews (PRISMA-ScR) (Tricco et al., 2018).

### Stage Ⅰ: identifying the research question

The research question of this review is “*To what extent have the items outlined in CONSORT-CHM Formula 2017 been adequately reported in RCTs for CHMF, and what is the overall standard of reporting quality observed subsequent to the release of CONSORT-CHM Formula 2017?*” In pursuit of this objective, a comprehensive search of RCTs related to CHMF, from both Chinese and international medical journals, will be gathered and meticulously evaluated using the CONSORT-CHM Formula checklist.

### Stage Ⅱ: study selection

#### Eligibility criteria

The PCC (Population, Concept, and Context) framework recommended by JBI will be applied to assess the eligibility of studies. This framework will equip our research with clear and meaningful inclusion and exclusion criteria (Peters et al., 2020). Studies are eligible for this review if they fulfill the following inclusion and exclusion criteria.

#### Population

Studies should be conducted targeting humans, and include patients with any diseases, or TCM syndromes. There will be no limitation on age, geography, and gender.

#### Concept

Prospective randomized controlled trials evaluating the clinical efficacy or safety of the CHMF will be included. An intervention will be categorized as a CHMF if it comprises more than one Chinese medicinal substance or extract of Chinese medicinal substances. Interventions synthesized with monomers are not defined as CHMF. Trials focusing on determining the optimal dosage, dosage form, processing methods, and composition of CHMF, as well as investigations into their valid indications, administration timing, routes, and application areas on the body, will be excluded. Additionally, trials investigating the combined effects of CHMF with other interventions will also be excluded. Secondary analyzed research, study protocols, reviews, and animal experiments will not be incorporated.

#### Context

The reports published in English or Chinese with a date range from 1 January 2018 to 8 June 2022 will be included, while studies without full text or repeated publications will be excluded.

#### Literature searching strategies

Systematical searches have been conducted across eight electronic databased: EBM Reviews, Allied and Complementary Medicine (AMED), Embase, Ovid-MEDLINE(R), Wanfang data, China National Knowledge Infrastructure (CNKI), VIP Chinese Medical Journal Database (VIP) and Chinese Biomedical Literature (CBM) database. The search for RCTs on CHMF was conducted on 8 June 2022. Thus, articles published between 1 January 2018 and 8 June 2022, with language restriction to English or Chinese were included. The detailed search strategy for each database is available in Supplementary Appendix S1.

#### Study screening

The process of article selection will be executed independently by two reviewers, following the recommendations of the PCC framework. All identified citations will be managed using EndNote X9 reference management software (https://endnote.com/), and any duplicate entries will be eliminated. After undergoing training provided by a senior researcher, the titles and abstracts will be meticulously reviewed to screen out the exclusion. Subsequently, the full text of potentially eligible articles will undergo a comprehensive review to ascertain their alignment with the inclusion criteria. For articles that do not meet the criteria for inclusion, detailed records will be maintained, documenting the specific reasons for their exclusion based on predetermined parameters. In instances where discrepancies arise, a consensus discussion will be facilitated involving a third, independent researcher. Details of the study selection process are shown in Supplementary Appendix S2.

### Stage III: extracting and charting the data

#### Background data collection

Data collection and management will be executed utilizing Microsoft Excel. Initial data entry and organization will be conducted by one reviewer, followed by thorough validation by an independent second reviewer. Firstly, a data extraction sheet will be designed to encompass a range of specific details. These details encompass fundamental information (e.g., name of the first author, year of publication, etc.), intervention information (e.g., name of CHMF, dosage form, etc.), study populations (e.g., name of diseases, TCM patterns (if any), etc.), aims of the study (i.e., efficacy, safety and both), methodology (e.g., blinding, randomization, etc.), outcome measures (i.e., TCM-related outcome measures and safety measures), and important results (e.g., sample size, harms, etc.). The data extraction form and rules are shown in Supplementary Appendix S3.

Secondly, the reviewers responsible for data extraction will initiate a pilot test on the initial 50 articles, utilizing the data extraction form to pinpoint any potential requirements for additional or modified criteria. As the data extraction process unfolds and new, relevant data types and themes emerge, the research team will actively refine the form. Any adjustments to the form will be made under the following circumstances: 1) If crucial information is omitted from the extraction form, the reviewers will supplement these data in the implementation of extraction. 2) To facilitate statistical analysis, the original data may be categorized. For instance, diseases might be categorized according to the disease categories outlined in the International Classification of Diseases 11th Revision for Mortality and Morbidity Statistics (ICD-11 MMS) version 02/2022 (World Health Organization, 2022). Thirdly, in case of discrepancies or disagreements among the reviewers, a collaborative discussion will be initiated to reach a resolution. If a consensus proves challenging to achieve, a third senior researcher will be consulted to facilitate a conclusive agreement.

#### Quality assessment with CONSORT-CHM formula 2017

In the CONSORT-CHM Formula 2017 checklist, 22 extended items have been specifically tailored for interventions involving CHMF. These items encompass aspects such as TCM pattern (if applicable), detailed description, quality control, and safety assessment. To enhance the efficiency of the assessment process, three senior researchers (XZ, CWC, and ZXB), who are also the authors of CONSORT-CHM Formula 2017, have developed a customized quality assessment form. In this form, each item of the CONSORT-CHM Formula 2017 has been reframed into one or more questions to facilitate a more intricate evaluation. For instance, the original reporting item 1b *“Illustration of the name and form of the formula used, and the TCM Pattern applied, if applicable”* has been subdivided into Q2 *“Whether the name of the CHM formula was reported in Abstract?”*, Q3 *“Whether the dosage form of the CHM formula was reported in Abstract?”*, and Q4 *“Whether the TCM Pattern was reported in Abstract?”*. The assessment form, which incorporates the 22 primary items of the CONSORT-CHM Formula 2017, is complemented by 42 sub-questions and is visually presented in Table 1.

**TABLE 1:** 42 sub-questions based on the CONSORT-CHM Formula 2017 checklist.

Section/topic	Extension items	Sub-questions for assessment
**Title, abstract, and keywords**	1a. Statement of whether the trial targets a TCM Pattern, a Western medicine–defined disease, or a Western medicine–defined disease with a specific TCM Pattern, if applicable	*Q1. Whether it reported that the trial targeted a specific TCM Pattern in “Title”?*
	1b. Illustration of the name and form of the formula used, and the TCM Pattern applied, if applicable	*Q2. Whether the name of the CHM formula was reported in “Abstract”?*
		*Q3. Whether the dosage form of the CHM formula was reported in “Abstract”?*
		*Q4. Whether the TCM Pattern was reported in “Abstract”?*
	1c. Determination of appropriate keywords, including “Chinese herbal medicine formula” and “randomized controlled trial”	*Q5. Whether the “Chinese herbal medicine formula” was presented in “Key word”?*
		*Q6. Whether “randomized controlled trials” was presented in “Key words”?*
**Introduction**	2a. Statement with biomedical science approaches and/or TCM approaches	*Q7. Whether the TCM background and explanation of the disease or the TCM Pattern was reported in “Background”?*
**Background and objectives**		
		*Q8. Whether the biomedical science explanation and/or TCM rationale about the CHM formula were reported in “Background”?*
	2b. Statement of whether the formula targets a Western medicine–defined disease, a TCM Pattern, or a Western medicine–defined disease with a specific TCM Pattern	*Q9. Whether the objective or hypotheses focused on the CHM formula in treatment of a Western medicine-defined disease, a TCM Pattern, or a Western medicine-defined disease with a specific TCM Pattern?*
**Methods**	4a. Statement of whether participants with a specific TCM Pattern were recruited, in terms of 1) diagnostic criteria and 2) inclusion and exclusion criteria. All criteria used should be universally recognized, or reference given to where detailed explanation can be found	*Q10. Whether the participants with a specific TCM Pattern were recruited, in terms of 1) diagnostic criteria and 2) inclusion and exclusion criteria, and whether all criteria used were universally recognized, or reference given to where detailed explanation can be found in “Methods”?*
**Participants**		
**Interventions**	5a-1. Name, source, and dosage form (e.g., decoctions, granules, powders)	*Q11. Whether the name of the CHM formula was reported in “Methods”?*
**5a. For fixed CHM formulas**		
		*Q12. Whether the source of the CHM formula was reported in “methods”?*
		*Q13. Whether the dosage form of the CHM formula was reported in “methods”?*
	5a-2. Name, source, processing method, and dosage of each medical substance. Names of substances should be presented in at least 2 languages: Chinese (Pinyin), Latin, or English. Names of the parts of the substances used should be specified	*Q14. Whether the name of each medical substance was reported in “Methods”?*
		*Q15. Whether the source of each medical substance was reported in “Methods”?*
		*Q16. Whether the processing method of each medical substance was reported in “Methods”?*
		*Q17. Whether the dosage of each medical substance was reported in “Methods”?*
	5a-3. Authentication method of each ingredient and how, when, where, and by whom it was conducted; statement of whether any voucher specimen was retained, and if so, where they were kept and whether they are accessible	*Q18.Whether the Authentication method of each ingredient was reported in “Methods”?*
	5a-4. Principles, rationale, and interpretation of forming the formula	*Q19. Whether the principles, rationale, and interpretation of forming the formula were reported?*
	5a-5. Reference(s) as to the efficacy of the formula, if any	*Q20. Whether the reference(s) as to the efficacy of the formula was presented?*
	5a-6. Pharmacologic study results of the formula, if any	*Q21. Whether the pharmacologic study results of the formula were presented?*
	5a-7. Production method of the formula, if any	*Q22. Whether the production method of the formula was reported?*
	5a-8. Quality control of each ingredient and of the product of the formula, if any. This would include any quantitative and/or qualitative testing method(s); when, where, how, and by whom these tests were conducted; whether the original data and samples were kept, and, if so, whether they are accessible	*Q23. Whether the quality control of each ingredient and of the product of the formula was conducted?*
	5a-9. Safety assessment of the formula, including tests for heavy metals and toxic elements, pesticide residues, microbial limit, and acute/chronic toxicity, if any. If yes, it should be stated when, where, how, and by whom these tests were conducted; if the original data and samples were kept; and, if so, whether they are accessible	*Q24. Whether the safety assessment of the formula was conducted?*
	5a-10. Dosage of the formula, and how the dosage was determined	*Q25. Whether the dosage of the formula was reported?*
		*Q26. Whether the treatment duration of the CHM formulas was reported in “Methods”?*
	5a-11. Administration route (e.g., oral, external)	*Q27. Whether the Administration route of the CHM formula was reported in “Methods”?*
**5b. For individualized CHM formulas**	5b-1. See recommendations 5a 1–11	*See Q11 to Q27*
	5b-2. Additional information: how, when, and by whom the formula was modified	*Q28. For trials with individualized CHM formulas, whether it reported how, when, and by whom the CHM formula was modified in “Methods”?*
**5c. For patent proprietary CHM formulas**	5c-1. Reference to publicly available materials, such as pharmacopeia, for the details about the composition, dosage, efficacy, safety, and quality control of the formula	*Q29. For trials with patent proprietary CHM formulas, whether the composition and dosage were reported in “Methods”?*
	5c-2. Illustration of the details of the formula, namely, 1) the proprietary product name (i.e., brand name), 2) name of manufacturer, 3) lot number, 4) production date and expiry date, 5) name and percentage of added materials, and 6) whether any additional quality control measures were conducted	*Q30. For trials with patent proprietary CHM formulas, whether the efficacy was reported in “Methods”?*
		*Q31. For trials with patent proprietary CHM formulas, whether the safety or quality control was reported in “Methods”?*
		*Q32. For trials with patent proprietary CHM formulas, whether the proprietary product name (i.e., brand name), name of the manufacturer, and lot number were reported in “Methods”?*
		*Q33. For trials with patent proprietary CHM formulas, whether the production date and expiry date were reported in “Methods”?*
	5c-3. Statement of whether the patent proprietary formula used in the trial is for a condition that is identical to the publicly available reference	*Q34. For trials with patent proprietary CHM formulas, whether the patent proprietary formula used in the trial is for a condition that is identical to the publicly available reference was stated?*
**5d. Control groups Placebo control**	5d-1. Name and amount of each ingredient	*Q35. For trials with placebo control, whether the name and amount of each ingredient of the placebo were reported in “Methods”?*
	5d-2. Description of the similarity of placebo with the intervention (e.g., color, smell, taste, appearance, packaging)	*Q36. For trials with placebo control, whether the similarity of placebo with the intervention (e.g., color, smell, taste, appearance, packaging) was reported in “Methods”?*
	5d-3. Quality control and safety assessment, if any	*Q37. For trials with placebo control, whether the quality control and safety assessment of the placebo were reported in “Methods”?*
	5d-4. Administration route, regimen, and dosage	*Q38. For trials with placebo control, whether the administration route, regimen, and dosage of the placebo were reported in “Methods”?*
	5d-5. Production information: where, when, how, and by whom the placebo was produced	*Q39. For trials with placebo control, whether the production information of the placebo was reported, including where, when, how, and by whom the placebo was produced?*
**Outcomes**	Illustration of outcome measures with Pattern in detail	*Q40. Whether the outcome measures included TCM indicators in “Outcome”?*
**Discussion**	Discussion of how the formula works on different TCM Patterns or diseases	*Q41. Whether any discussion of how the formula works on different TCM Patterns or diseases was reported in “Discussion” ?*
**Generalizability**		
**Interpretation**	Interpretation with TCM theory	*Q42. Whether any interpretation with TCM theory was reported in “Discussion”?*

TCM: traditional Chinese medicine; CHM: chinese herbal medicine.

In the evaluation process, each item will receive “1 point” for an item only if all the pertinent information details specified within that CONSORT-CHM Formula item have been adequately reported. Conversely, “0 point” will be assigned if the required information is only partially disclosed or entirely absent. Instances labeled as “Not Applicable” (NA) signify that a specific item or sub-question is not pertinent to a given RCT. A detailed outline of the standards operating procedure (SOP) for conducting quality assessment, drafted by three senior researchers (XZ, CWC, and ZXB), is presented in Supplementary Appendix S4. In the SOP, we pre-defined the criteria for scoring each question, outlining circumstances warranting a score of 1 point, conditions justifying a score of 0 points, and whether the item is subject to an “NA” designation. For example, regarding three circumstances are provided in SOP, including 1) “1 score” is considered if the specific name of the CHM formula was reported in the section of “Methods,” such as “*Danggui Shaoyao San*.” In addition, the name representing the treatment principle of CHMF is also accepted, such as “Harmonizing and Descending Reversal Formula”. 2) “0 score” is assigned if the authors fail to specify the exact name of the CHM formula in the “Methods” section, reporting only general terms such as “Chinese herbal medicines,” “CHM formula,” or “TCM therapeutics,”; It is important to note that if the specific name of the studied CHM Formula is mentioned in the title, abstract, or introductions, aside from the “Methods” section, it will still be scored as “0”. 3) “NA” does not apply to this item.

To ensure a thorough understanding of the CONSORT-CHM Formulas 2017 and its dedicated assessment form, each reviewer will thoroughly examine the explanatory documents before initiating the quality assessment. A pilot test, involving the evaluation of 100 randomly selected articles, will be conducted. Reviewers who have attained a correctness rate of at least 70% will be included in the work of formal quality assessment. In the process of quality assessment, if reviewers encounter any issues, especially those pertaining to the actual content or features of the article that fall beyond the scope of the assessment rules pre-designed in the SOP, the reviewers are required to promptly propose the question and to communicate with the collaborative team. Supplements are allowed in the SOP documents. Besides, in case of an error made by a reviewer, the mistake and its corresponding corrections will be communicated to the entire collaborative team to ensure consistency. The SOP will be updated based on recommendations from senior researchers (XZ, CWC, and ZXB), insights from the pilot assessment results, and feedback from the reviewers. It should be noted that all articles included in this review will be assessed by two independent reviewers. Cohen’s kappa will be used to identify the level of agreement between the two reviewers. Any problems or ambiguities that arise will be resolved with the consultation of the third senior researcher. In the results of this study, we will present the SOP document again, accompanied by scoring examples. Any disparities between the implementation and this protocol will be reported in the final manuscript with explanations provided for clarity.

### Stage IⅤ: collating, summarizing, and reporting results

The extracted data will be systematically collated and summarized in accordance with the analytical framework outlined in the review. Following this, a narrative summary of findings will be crafted, drawing upon background data and guided by the research questions and study objectives. To assess reporting quality characteristics, a quantitative summary will be executed, calculating frequencies and proportions for each item/sub-question found in the included articles. These results will be presented in tabulated form. Additionally, a descriptive narrative will be provided to outline reporting quality trends across RCTs, accompanied by thematic insights derived from the accumulated studies. Moreover, subgroup analysis will be conducted to investigate factors influencing the reporting quality of RCTs, where applicable. Potential contributing variables such as formula types, publication years, and distributions of study countries will be considered in these analyses. The results of these analyses will be presented comprehensively. To enhance understanding, illustrative examples featuring well-documented reports will be compiled. These examples will serve to demonstrate the practical application of CONSORT-CHM Formula 2017 within the realm of implementation research, offering tangible instances of effective implementation.

Descriptive statistics, such as frequencies and proportions, will be employed to summarize the characteristics of the included studies. Concerning the compliance rate (CR, calculated as CR = n/N *100%) of reporting, the count of “not applicable” instances will be excluded from the calculation. Specifically, “n” represents the number of RCTs categorized as “Fully reported,” while “N” denotes the total included RCTs minus the count of instances labeled as “not applicable.” For subgroup comparisons, Fisher’s exact test will be utilized, if applicable. Statistical significance will be established at *p* < 0.05. Additionally, where appropriate, binary logistic regression will be considered to assess the probabilities as a function of determined explanatory factors influencing reporting quality. The above statistical analysis will be conducted using Microsoft Excel 2016 and SPSS 23.0 (IBM Corp.).

## Discussion

The CONSORT-CHM Formula 2017 has established a reporting standard for RCTs involving CHMF interventions; however, its adherence and improvements for the reporting remain unclear. This review aims to conduct a thorough analysis of RCTs with CHMF interventions in recent 5 years, without limitations on specific diseases. Consequently, this undertaking seeks to comprehensively explore, map, and synthesize the characteristics, reporting quality, and completeness of RCTs involving CHMF since the release of the CONSORT-CHM Formula in 2017. The results of this review will serve a dual purpose. Firstly, it will identify areas of knowledge gaps, contributing to the improvement of reporting transparency and the improvements of CHMF-related publications. Secondly, it will shed light on the need for spread promotions to the CONSORT-CHM Formula 2017, especially for specific enhancement items for the actual application.

Review articles, as a common study type, are distinguished by their systematic and replicable methodology, leading to the derivation of overarching theoretical insights (Siddaway et al., 2019). The strengths of this study include the stringent adherence to Arksey and O’Malley’s scoping review framework (Arksey and O’Malley, 2005), coupled with the application of a comprehensive systematic search strategy across eight bibliographic databases to identify eligible articles. Furthermore, the data screening, extraction, and assessment of each article will be independently conducted by two independent reviewers. All reviewers are required to attend standard training (provided by senior researchers) and pass the pilot testing. A significant highlight of this review lies in the formulation of a specific SOP document for reporting assessment to ensure repetition of scoring.

However, certain limitations are inherent to this study. Firstly, the focus is not exclusively centered on RCTs related to specific diseases but rather on populations undergoing CHMF therapies. This broad search approach may potentially yield a considerable number of redundant texts or publications. Secondly, despite our commitment to performing a comprehensive search, studies published in languages other than Chinese or English and not within the search period might inadvertently be excluded.

## Conclusion

This proposed review aims to conduct a comprehensive assessment of the adherence to CONSORT-CHM Formula 2017 in published RCTs involving CHMF over the past 5 years, thereby mapping and synthesize the landscape of RCTs in the CHMF domain, shedding light on their study characteristics, reporting quality, and compliance after the release of CONSORT-CHM Formula 2017. Ultimately, we anticipate that this review will identify the current reporting characteristics and quality of CHMF interventional studies, providing valuable insights into the impact of CONSORT-CHM Formula 2017. The results obtained may offer suggestions for the future application or promotion of the guideline.
