# Supplementary material for: Evaluation of compliance of CONSORT-CHM formula 2017 in randomized controlled trials of Chinese herbal medicine formulas: protocol of a five-year review
Source: Front Pharmacol. 2024 Feb 23;15:1287262. doi: 10.3389/fphar.2024.1287262 (PMC10921883; doi:10.3389/fphar.2024.1287262)
Supplement: Supplementary file 1 [file DataSheet1.PDF]

Appendix files

Contents

Appendix file 1 Search strategies for literature review. 2
Appendix file 2 Overall flow chart of the search and selection process. 4
Appendix file 3 The data extraction form and rules. 5
Appendix file 4 The detailed outline of the standards operating procedure (SOP) for conducting quality assessment 9

Appendix file 1 Search strategies for literature review.

1. Ovid

Set	Search term
1	random$.tw.
2	factorial$.tw.
3	(crossover$ or cross over$ or cross-over$).tw.
4	placebo$.tw.
5	single blind.mp.
6	double blind.mp.
7	triple blind.mp.
8	(singl$ adj blind$).tw.
9	(double$ adj blind$).tw.
10	(tripl$ adj blind$).tw.
11	assign$.tw.
12	allocat$.tw.
13	randomized controlled trial/
14	or/1-13
15	((single#entity or single) adj3 (component or drug$ or herb$)).mp.
16	(compound prescription$ or herbal mixture or Fufang).mp.
17	(Chinese Medicine Patent Prescription or proprietary Chinese medicines).mp.
18	(Chinese patent adj3 (medicine or drug$)).mp.
19	(Chinese adj3 (patent or proprietary) adj3 (medicine or drug$)).mp.
20	(Chinese adj2 (patent or proprietary) adj2 (medicine or drug$ or prescription$)).mp.
21	or/15-20
22	14 and 21
23	limit 22 to yr="2018 -Current" [Limit not valid in DARE; records were retained]

2. VIP database

(M=临床试验+M=随机+M=对照+M=随机试验+M=随机对照试验+M=临床研究+M=半随机) AND (中医药+M=草药+M=中药+M=复方+M=汤剂+M=中成药+M=注射剂+M=加减方+M=外用中药) AND 年份: 2018-2022

3. Wanfang database

(题名: (临床试验 or 随机 or 对照 or 随机试验 or 随机对照试验 or 临床研究 or 半随机)) and (题名或关键词:(中医药 or 草药 or 中药 or 复方 or 汤剂 or 中成药 or 注射剂 or 加减方 or 外用中药)) and Date:2018-2022

4. **CNKI database**

(SU="临床试验" or SU="随机" or SU="对照" or SU="随机试验" or SU="随机对照试验" or SU="临床研究" or SU="半随机") AND (SU="中医药" or SU="草药" or SU="中药" or SU="复方" or SU="汤剂" or SU="中成药" or SU="注射剂" or SU="加减方" or SU="外用中药) AND (发表时间：2018-01-01-2022-06-08)

5. **CBM database**

((("中医药"[标题] OR "草药"[标题] OR "中药"[标题] OR "复方"[标题] OR "汤剂"[标题] OR "中成药"[标题] OR "注射剂"[标题] OR "加减方"[标题] OR "外用中药"[标题]) OR ("中医药"[摘要] OR "草药"[摘要] OR "中药"[摘要] OR "复方"[摘要] OR "汤剂"[摘要] OR "中成药"[摘要] OR "注射剂"[摘要] OR "加减方"[摘要] OR "外用中药"[摘要])) AND ("临床试验"[标题] OR "随机"[标题] OR "对照"[标题] OR "随机试验"[标题] OR "随机对照试验"[标题] OR "临床研究"[标题] OR "半随机"[标题])) AND 2018-2022[日期]

Appendix file 2 Overall flow chart of the search and selection process.

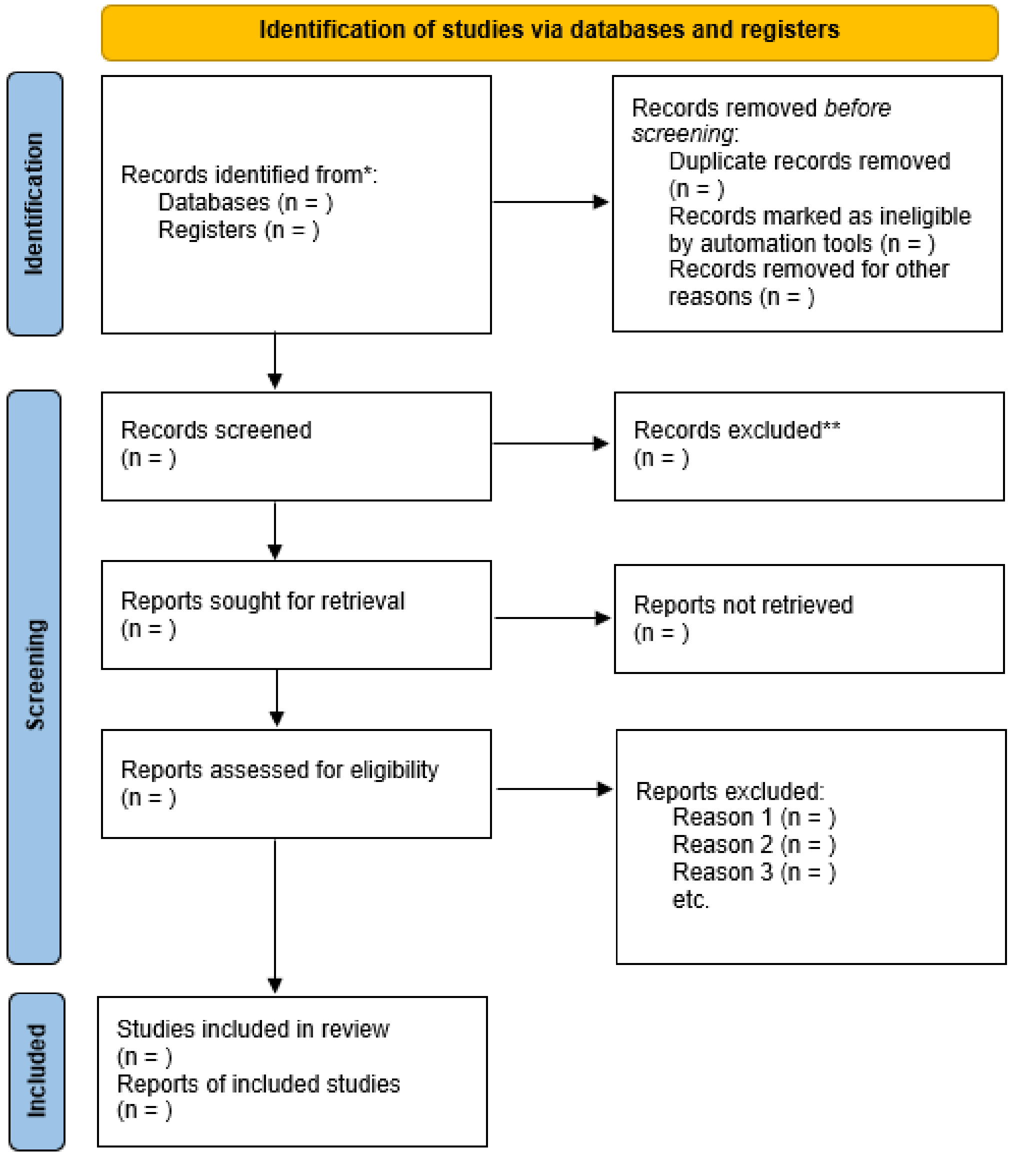

* From: Page MJ, McKenzie JE, Bossuyt PM, Boutron I, Hoffmann TC, Mulrow CD, et al. The PRISMA 2020 statement: an updated guideline for reporting systematic reviews. BMJ 2021;372:n71. doi: 10.1136/bmj.n71

Appendix file 3 The data extraction form and rules.

No.	Items	Rules for Data extraction
**Part 1**	**Information of included articles, journals, and corresponding authors**	
**1**	Article ID	The exported article's information includes the record number, author, title, and year of publication. Select the combination of the last name of first author and the year of publication as the article ID.
**2**	Year of publication	Extract via EndNote 20.
**3**	Study title	Extract via EndNote 20.
**4**	Language	Classify as English or Chinese.
**5**	Number of authors	Extract via EndNote 20.
**Part 2**	**Study Design**	
**6**	Trial objective	Classify into three categories: 1=Assessing the clinical efficacy of CHM formulas, 2=Assessing the safety of CHM formulas, or 3=Assessing the clinical efficacy and satefty of CHM formulas.
**7**	Number of recruitment sites	Classify into two categories: 1=Single-centre or 2=Multiple centre. Number of recruitment sites will be extracted from the methods section in full text.
**8**	Nation of recruitment sites	Record the nation of recruitment sites in the study. Nation of recruitment sites will be extracted from the methods section in full text.
**9**	Number of arms	Classify into two categories: 1=Two arms or 2=Multiple arms. Number of arms will be extracted from the methods section in full text.
**10**	Type of randomization	The method by which participants were randomly assigned can be categorized into two types: 1=Simple randomization or 2=Others. Other types of randomization include stratified randomization, central randomization, and minimization randomization.
**11**	Blinding	Classify into three categories: 0=Open label or 1=Blinding
**Part 3**	**Participants**	
**12**	Type of disease/symptoms	Diseases/symptoms reported in the study are classified according to the International Classification of Diseases 11th Revision (ICD-11) (https://icd.who.int/browse11/l-m/en), and

		record the number of the reported disease in ICD-11.<br>The total number of diseases/symptoms will exceed the number of included studies because some diseases/symptoms may fall into more than one category.<br>✧ e.g., “11” (The disease coded as “11” in the ICD-11 is categorized under circulatory system disorders)
**13**	Diagnosis of TCM patterns	The classification consists of two categories: 1=There was a diagnosis for TCM patterns or 2=There was not a diagnosis for TCM patterns. The diagnosis of TCM patterns will be extracted from the methods section in full text.
**14**	Total sample size	Record the initial total sample size as reported in the article, without considering any subsequent updates in the trial.
**15**	Gender of recruited participants	Record the genders of recruited participants in the trials, and Classify into three categories: 1=Only male, 2=Only female, or 3=Both male and female. This item was extracted from the methods section in full text.
**Part 4**	**Interventions**	
**16**	Type of CHM formulas	Classify into three categories according to the CONSORT-CHM Formulas 2017: 1=Fixed CHM formulas, 2=Individualized CHM formulas, or 3=Patent proprietary CHM formulas. Two or three types of CHM formulas may be applied in one trial, and multiple selection is allowed in this item.
**17**	Treatment duration	Classify into five categories: 1=Equal to or less than 1 week, 2=More than 1 week and equal to or less than 4 weeks, 3=More than 4 weeks and equal to or less than 12 weeks, 4=More than 12 weeks and equal to or more than 52 weeks, or 5=More than 52 weeks. This item will be extracted from the methods section in full text.
**18**	Following period	Classify into five categories: 1=Equal to or less than 1 week, 2=More than 1 week and equal to or less than 4 weeks, 3=More than 4 weeks and equal to or less than 12 weeks, 4=More than 12 weeks and equal to or more than 52 weeks, or 5=More than 52 weeks. This item will be extracted from the methods section in full text.
**Part 5**	**Comparison**	
**19**	Type of controls	Classify into four categories: 1=Including placebo as control, 2=Including positive controls, 3=Add-on design, or 4= Including blank or healthy as control.

		The total number of control types will exceed the number of included studies because several studies have more than two arms.
**Part 6**	**Outcomes**	
**20**	Primary and secondary outcomes	Classify into two categories: 1=Reported primary and secondary outcomes or 2=Not set primary or secondary outcomes.
**21**	TCM-related outcomes	Classify into four categories: 0= No TCM-related outcomes, 1=TCM patterns as outcomes, 3=TCM symptoms as outcomes, or 4=Both TCM patterns and symptoms as outcomes.
**22**	Safety outcomes	Classify into two categories: 0=No safety outcomes or 2=Including safety outcomes.
**Part 7**	**Harms**	
**23**	Adverse effects (AEs) reported	Classify into two categories: 0=No AE was reported, or 1=AE was reported. AEs can be identified in the section of results of the article. Not reported (NR) was also considered: if it was reported that safety outcomes would be measured in the section of methods, but no information about AEs was reported in the results section.
**24**	Groups the AEs occurred	Classify into five categories: 0=No AE was reported, 1=AEs occurred in intervention groups, 2=AEs occurred in controlled groups, 3=AEs both occurred in intervention groups and controlled groups, or 4=AEs occurred, but not be specified in which group.
**25**	Classification of AEs in the intervention group	According to the National Medical Products Administration in China (https://www.nmpa.gov.cn/xxgk/kpzhsh/kpzhshyp/20110705171101327.html), we refer to their explanation and Classify the AEs extracted from the included studies into five categories: 0=There was no AE in the experimental group, 1= Skin damage (e.g., rash, itching, etc.), 2=Digestive system damage (e.g., nausea, vomiting, diarrhea, constipation, abnormal liver function, etc.), 3=Urinary system damage (e.g., hematuria, renal dysfunction, etc.), or 4=Systemic damage (e.g., anaphylactic shock, fever, etc.).<br>Several types of categories will be recorded for one study because more than one type of AEs will be extracted.
**26**	Classification of AEs in the controlled group	The same as Item 23. Only one category is different, 0 refers to that there is no AE in the controlled group.
**27**	Reason of AEs	Classify into 8 categories: 0=No AE was reported, 1=because of CHM formulas, 2=Because

		of controlled interventions, 3=Because of the interaction of CHM formulas and western medicine interventions, 4=Because of the intervention, 5=Because of the individual constitutions of participants and not related to interventions, 6=Because of unknown reason, or 7=No reason was reported.
**28**	Times of AEs	Classify as 6 categories: 0=no AE was reported, 1=AEs occurred during the intervention, 2=AEs occurred at the end of intervention, 3=AEs occurred during the follow-up, 4=AEs occurred at the end of follow-up, or 5=AEs occurred, but not be specified the time of AEs.
**Part 8**	**Funding, registration, and protocol**	
**29**	Funding reported	Classify into three categories: 0=The study reported “no funding” involved, 1=The study was funded.

* TCM: traditional Chinese medicine; CHM: Chinese herbal medicine; CONSORT-CHM Formulas 2017: CONSORT Extension for Chinese Herbal Medicine Formulas 2017; AE: adverse effect

* It will be recorded as “NR (not reported)” if it did not report the item in original article.

Appendix file 4 The detailed outline of the standards operating procedure (SOP) for conducting quality assessment

Section/topic	Extension items	Questions for assessment	Definition of Fully reported (scored as 1), Partially or not reported (scored as 0), and Not applicable (NA)
**Title, abstract, and keywords**	Statement of whether the trial targets a TCM Pattern, a Western medicine–defined disease, or a Western medicine–defined disease with a specific TCM Pattern, if applicable	Q1. Whether it reported that the trial targeted a specific TCM Pattern in "Title"?	"Full reported" is considered if (1) a specific TCM Pattern was reported in Title when the trial targeted a TCM Pattern or a Western medicine–defined disease with a specific TCM Pattern; or (2) "treatment based on syndrome differentiation" were reported in Title when the trial targeted more than one TCM Patterns or a Western medicine-defined disease with several TCM patterns. The sub-question was identified from Title and Methods section in full text at the same time.<br>"Partially or not reported" is considered if no TCM Pattern was reported in Title when the trial targeted a/several TCM Pattern(s) in Methods section in full text.<br>"Not applicable" is considered if the trial targeted a Western medicine-defined disease.
	Illustration of the name and form of the formula used, and the TCM Pattern applied, if applicable	Q2. Whether the name of the CHM formula was reported in "Abstract"?	"Full reported" is considered if the name of the CHM formula was reported in Abstract. Including (1) the name of fixed CHM formulas; (2) "individualized formulas" "hospital preparation" when it refers to individualized CHM formulas; or (3) trade name of patent proprietary CHM formulas.<br>"Partially or not reported" is considered if (1) the name of the CHM formula was Partially or not reported in Abstract, such as "traditional Chinese medicines", "Chinese herbal medicine formulas", "integrative Chinese and Western medicine", or the name of treatment (e.g. "self-regulated CHM formula", "Yiqi Huoxue therapy", or "wish with traditional chinese medicines"); or (2) there was no Abstract.<br>"Not applicable" does not apply to this item.
		Q3. Whether the dosage form of the CHM formula was reported in "Abstract"?	"Full reported" is considered if the dosage form of the CHM formula was reported in Abstract. Including (1) it reported the specific dosage form of CHM formulas, such as "pill", "granule", "tablet", etc; (2) name of patent proprietary CHM formulas can indicate the dosage forms, such as "Danshen injection",

			“Niuhuang Jiedu Tablet”, or Yiqi Yangblood oral liquid”, etc; (3) the dosage form can be judged based on the specified and detailed production process of CHM formulas; or (4) name of CHM formulas include “decoction” “pill”, and it can be judged as decoction or pills based on the production process or administration routes of CHM formulas.<br>“Partially or not reported” is considered if (1) the dosage form of the CHM formula was Partially or not reported in Abstract, and the production process is not specific with summary words “水煎服/打散/研末”; or (2) there was no abstract.
		Q4. Whether the TCM Pattern was reported in “Abstract”?	“Full reported” is considered if (1) a specific TCM Pattern was reported in Abstract when the trial targeted a TCM Pattern or a Western medicine–defined disease with a specific TCM Pattern; or (2) “treatment based on syndrome differentiation” were reported in Abstract when the trial targeted more than one TCM Patterns or a Western medicine-defined disease with several TCM patterns. The sub-question was identified from Abstract and Methods section in full text at the same time.<br>“Partially or not reported” is considered if no TCM Pattern was reported in Abstract when the trial targeted a/several TCM Pattern(s) in Methods section in full text.<br>“Not applicable” is considered if the trial targeted a Western medicine-defined disease.
	Determination of appropriate keywords, including “Chinese herbal medicine formula” and “randomized controlled trial”	Q5. Whether the “Chinese herbal medicine formula” was presented in “Key word”?	“Full reported” is considered if Keywords included the “Chinese herbal medicine formula”.<br>“Partially or not reported” is considered if the Keywords did not include “Chinese herbal medicine formula”; or there were no Keywords.<br>“Not applicable” does not apply to this item.
		Q6. Whether “randomized controlled trials” was presented in “Key words”?	“Full reported” is considered if Keywords included “randomized control trials” or “RCT”.<br>“Partially or not reported” is considered if the Keywords did not include “randomized control trials” or “RCT”; or there were no Keywords.<br>“Not applicable” does not apply to this item.

**Introduction Background and objectives**	Statement with biomedical science approaches and/or TCM approaches	Q7. Whether the TCM background and explanation of the disease or the TCM Pattern was reported in "Background"?	"Full reported" is considered if the TCM background and explanation of the disease or TCM Pattern were reported in Background.<br>"Partially or not reported" is considered if no TCM interpretation about the disease or TCM Pattern was reported in Background.<br>"Not applicable" does not apply to this item.
		Q8. Whether the biomedical science explanation and/or TCM rationale about the CHM formula were reported in "Background"?	"Full reported" is considered if (1) the biomedical science explanation and/or TCM rationale about the CHM formula were reported in Background; (2) the research foundation or application history of CHM formulas were reported in Background.<br>"Partially or not reported" is considered if no introduction of the CHM formula was reported in Background.<br>"Not applicable" does not apply to this item.
	Statement of whether the formula targets a Western medicine–defined disease, a TCM Pattern, or a Western medicine–defined disease with a specific TCM Pattern	Q9. Whether the objective or hypotheses focused on the CHM formula in treatment of a Western medicine-defined disease, a TCM Pattern, or a Western medicine-defined disease with a specific TCM Pattern?	"Full reported" is considered if (1) a specific TCM Pattern was reported in objective or hypotheses when the trial targeted a TCM Pattern or a Western medicine–defined disease with a specific TCM Pattern; or (2) "treatment based on syndrome differentiation" were reported in objective or hypotheses when the trial targeted more than one TCM Patterns or a Western medicine-defined disease with several TCM patterns. The sub-question was identified from objective or hypotheses and Methods section in full text at the same time.<br>"Partially or not reported" is considered if no TCM Pattern was reported in objective or hypotheses when the trial targeted a/several TCM Pattern(s) in Methods section in full text.<br>"Not applicable" is considered if the trial targeted a Western medicine-defined disease.
**Methods Participants**	Statement of whether participants with a specific TCM Pattern were recruited, in terms of 1) diagnostic criteria and 2) inclusion and exclusion criteria. All criteria used should be universally recognized, or reference given to where detailed explanation can be found.	Q10. Whether the participants with a specific TCM Pattern were recruited, in terms of 1) diagnostic criteria and 2) inclusion and exclusion criteria, and whether all criteria used were universally recognized, or reference given to where detailed	"Full reported" is considered if the participants with a specific TCM Pattern were recruited, in terms of *a.* diagnostic criteria and *b.* inclusion and exclusion criteria. And all criteria used were universally recognized; or reference was given to where a detailed explanation can be found; or a detailed description of the TCM Pattern was provided, such as the symptoms, assessment measures, or scales.<br>"Partially or not reported" is considered if (1) there were no eligibility criteria for participants; (2) there were no eligibility criteria for TCM Pattern, but the trial targeted

		explanation can be found in “Methods”?	a TCM Pattern or a Western medicine–defined disease with a specific TCM Pattern; or (3) there were no recognized criteria, reference, or detailed description of the eligibility criteria for TCM Pattern in Methods section.<br>“Not applicable” is considered if the trial targeted a Western medicine-defined disease.
**Interventions 5a. For fixed CHM formulas**	1. Name, source, and dosage form (e.g., decoctions, granules, powders)	Q11. Whether the name of the CHM formula was reported in “Methods”?	“Full reported” is considered if the name of the CHM formula was reported in Method. Every formula should be reported the name if more than one CHM formula was applied in the trial.<br>“Partially or not reported” is considered if (1) it did not report the specific name of the CHM formula, only referred to the “Chinese herbal medicines”, “CHM formula”, or names of TCM therapeutics; or (2) it did not report all names of formulas when several CHM formulas were applied in the trial.<br>“Not applicable” does not apply to this item.
		Q12. Whether the source of the CHM formula was reported in “methods”?	“Full reported” is considered if (1) it reported where the CHM formula originated from, such as the classic TCM books, researcher articles, or adapted from a famous classic prescription; (2) it reported who created the CHM formula; or (3) the trial applied a self-made formula along with its detailed explanations for basic formulating principles. If several formulas were applied in the trial, it should report the resources for every formula.<br>“Partially or not reported” is considered if readers can not know about the source of the CHM formula; or it did not report the sources for every formula when several formulas were applied in the trial.<br>“Not applicable” does not apply to this item.
		Q13. Whether the dosage form of the CHM formula was reported in “methods”?	“Full reported” is considered if the dosage form of the CHM formula was reported in Abstract. Including (1) it reported the specific dosage form of CHM formulas, such as “pill”, “granule”, “tablet”, etc; (2) name of patent proprietary CHM formulas can indicate the dosage forms, such as “Danshen injection”, “Niuhuang Jiedu Tablet”, or Yiqi Yangblood oral liquid”, etc; (3) the dosage form can be judged based on the specified and detailed production process of CHM formulas; or (4) name of CHM formulas include “decoction” “pill”, and it can be judged as decoction or

			pills based on the production process or administration routes of CHM formulas.<br>"Partially or not reported" is considered if (1) the dosage form of the CHM formula was Partially or not reported in Abstract, and the production process is not specific with summary words "水煎服/打散/研末"; or (2) there was no abstract.
	2. Name, source, processing method, and dosage of each medical substance. Names of substances should be presented in at least 2 languages: Chinese (Pinyin), Latin, or English. Names of the parts of the substances used should be specified.	Q14. Whether the name of each medical substance was reported in "Methods"?	"Full reported" is considered if the name of each medical substance was reported in Methods.<br>"Partially or not reported" is considered if (1) it did not report the name of each medical substance, including there is an "etc." at the end of the name of the last substance.; (2) it did not report all ingredients of every formula when more than one formula was applied in the trial.<br>"Not applicable" is considered if only the patent proprietary CHM formula was applied in the trial.
		Q15. Whether the source of each medical substance was reported in "Methods"?	"Full reported" is considered if the source of the medical substance was reported in Methods. Including (1) At least the sources of one substance were reported; (2) the sources of some TCM substances are included in their name, such as "Hang Baiju" which means the white Chrysanthemum that is made in Hangzhou, Zhejiang province.<br>"Partially or not reported" is considered if it did not report the source of the medical substance.<br>"Not applicable" is considered if only the patent proprietary CHM formula was applied in the trial.
		Q16. Whether the processing method of each medical substance was reported in "Methods"?	"Full reported" is considered if the processing method of the medical substance was reported in Methods. Including (1) At least the processing methods of one substance were reported; (2) the processing methods of some TCM substances are included in their name, such as "Cu Banxia" which means the Rhizoma Pinelliae that has been prepared with vinegar; (3) it did not report the specific processing methods of substances, but the production criteria was provided, such as "the Rhizoma Pinelliae meet the criterion that was described in *Chinese Pharmacopoeia*".<br>"Partially or not reported" is considered if it

			did not report the processing methods of the medical substance.<br>"Not applicable" is considered if only the patent proprietary CHM formula was applied in the trial.
		Q17. Whether the dosage of each medical substance was reported in "Methods"?	"Full reported" is considered if (1) the dosage of each medical substance was reported in Methods, the units can be gram, tael, litre, or specific number; (2) it reported the ratio of the medical substances.<br>"Partially or not reported" is considered if the dosage of each medical substance was Partially or not reported in Methods; or it did not report the dosages of each substance for every formula when several formulas were applied in the trial.<br>"Not applicable" is considered if only the patent proprietary CHM formula was applied in the trial.
	3. Authentication method of each ingredient and how, when, where, and by whom it was conducted; statement of whether any voucher specimen was retained, and if so, where they were kept and whether they are accessible	Q18.Whether the Authentication method of each ingredient was reported in "Methods"?	"Full reported" is considered if it reported the authentication method of each ingredient and how, when, where, and by whom it was conducted.<br>"Partially or not reported" is considered if it did not report the authentication method of each ingredient.<br>"Not applicable" is considered if only the patent proprietary CHM formula was applied in the trial.
	4. Principles, rationale, and interpretation of forming the formula	Q19. Whether the principles, rationale, and interpretation of forming the formula were reported?	"Full reported" is considered if the principles, rationale, and interpretation of forming the formula were reported.<br>"Partially or not reported" is considered if the principles, rationale, and interpretation of forming the formula were Partially or not reported.<br>"Not applicable" is considered if only the patent proprietary CHM formula was applied in the trial.
	5. Reference(s) as to the efficacy of the formula, if any	Q20. Whether the reference(s) as to the efficacy of the formula was presented?	"Full reported" is considered if reference(s) as to the efficacy of the formula was presented.<br>"Partially or not reported" is considered if reference(s) as to the efficacy of the formula was not presented.<br>"Not applicable" does not apply to this item.
	6. Pharmacologic study results of the formula, if any	Q21. Whether the pharmacologic study	"Full reported" is considered if pharmacologic study results of the CHM formula were reported.

		results of the formula were presented?	"Partially or not reported" is considered if the pharmacologic study results of the formula were not reported.<br>"Not applicable" does not apply to this item.
	7. Production method of the formula, if any	Q22. Whether the production method of the formula was reported?	"Full reported" is considered if (1) the production methods of the formula were reported; (2) it reported the formulas were produced by hospital, pharmacy, or pharmaceutical company; or (3) the production criteria with reference(s) of the CHM formula were reported in Methods.<br>"Partially or not reported" is considered if the production methods were Partially or not reported in Methods.<br>"Not applicable" is considered if only the patent proprietary CHM formula was applied in the trial.
	8. Quality control of each ingredient and of the product of the formula, if any. This would include any quantitative and/or qualitative testing method(s); when, where, how, and by whom these tests were conducted; whether the original data and samples were kept, and, if so, whether they are accessible.	Q23. Whether the quality control of each ingredient and of the product of the formula was conducted?	"Full reported" is considered if the quality control of each ingredient and of the product of the formula was conducted in the trial. This would include any quantitative and/or qualitative testing method(s); when, where, how, and by whom these tests were conducted; whether the original data and samples were kept, and, if so, whether they are accessible.<br>"Partially or not reported" is considered if the quality control of each ingredient and of the product of the formula was partially or not reported in the trial.<br>"Not applicable" is considered if only the patent proprietary CHM formula was applied in the trial.
	9. Safety assessment of the formula, including tests for heavy metals and toxic elements, pesticide residues, microbial limit, and acute/chronic toxicity, if any. If yes, it should be stated when, where, how, and by whom these tests were conducted; if the original data and samples were kept; and, if so, whether they are accessible.	Q24. Whether the safety assessment of the formula was conducted?	"Full reported" is considered if the conduction of safety assessment was reported in the trial. If the safety assessment of the formula was reported, it should include the tests for heavy metals and toxic elements, pesticide residues, microbial limit, and acute/chronic toxicity. And it should be stated when, where, how, and by whom these tests were conducted; if the original data and samples were kept; and, if so, whether they are accessible.<br>"Partially or not reported" is considered if the safety assessment was partially or not reported in the trial.<br>"Not applicable" is considered if only the patent proprietary CHM formula was applied in the trial.

	10. Dosage of the formula, and how the dosage was determined	Q25. Whether the dosage of the formula was reported?	"Full reported" is considered if (1) the dosage of the formula was reported in Methods.; (2) it reported the dosage of each medical substance of the formulas; (3) it reported the reference(s) on which the dosage was based; or (4) it reported the name of the manufacturer and lot number of the formula if only patent proprietary CHM formulas were applied in the trial.<br>"Partially or not reported" is considered if the detailed dosage information of the formula was partially (at least the name or dosage of one substance was not reported) or not reported in Methods.<br>"Not applicable" does not apply to this item.
		Q26. Whether the treatment duration of the CHM formulas was reported in "Methods"?	"Full reported" is considered if the treatment duration of the formula was reported in Methods, including (1) the specific time or (2) the number of cycles (menstrual cycle or treatment cycle)<br>"Partially or not reported" is considered if (1) the treatment duration of the formula was not reported; (2) readers can not learn about the treatment duration, such as the description "treat until the pain goes away"; or (3) it did not report the treat duration of each formula when several formulas were applied in the trial.<br>"Not applicable" does not apply to this item.
	11. Administration route (e.g., oral, external)	Q27. Whether the Administration route of the CHM formula was reported in "Methods"?	"Full reported" is considered if the administration route of the formula was reported in Methods. We do not infer the administration routes according to the dosage forms or production methods of fomulas.<br>"Partially or not reported" is considered if (1) the administration route of the formula was not reported in Methods; or (2) it did not report the treat duration of each formula when several formulas were applied in the trial.<br>"Not applicable" does not apply to this item.
**5b. For individualized CHM formulas**	1. See recommendations 5a 1–11<br>2. Additional information: how, when, and by whom the formula was modified	Q28. For trials with individualized CHM formulas, whether it reported how, when, and by whom the CHM formula was modified in "Methods"?	"Full reported" is considered if it reported how, when, and by whom the CHM formula was modified when the individualized CHM formula was used as the intervention in the trial. If more than one formulas was applied in the trial, it should reported the specific medical substances of each formula.<br>"Partially or not reported" is considered if it did not report the modification details of the

			individualized CHM formula in the trial, including (1) it described "modify the ingredients of formula based on different TCM patterns" without specific methods and medicines for modification; (2) it described "modify the ingredients of formula based on different symptoms" without specific methods and medicines for modification; (3) it described "modify the formulas based on different TCM patterns" without specific ingredients of each formula.<br>"Not applicable" is considered if (1) no individualized CHM formula was applied in the trial; (2) more than one fixed CHM formulas was applied in the trial, such as it applied the fixed CHM formula A before menstruation and the fixed CHM formula B after menstruation.
**5c. For patent proprietary CHM formulas**	1. Reference to publicly available materials, such as pharmacopeia, for the details about the composition, dosage, efficacy, safety, and quality control of the formula	Q29. For trials with patent proprietary CHM formulas, whether the composition and dosage were reported in "Methods"?	"Full reported" is considered if (1) the composition and dosage of the patent proprietary CHM formula were reported; (2) readers can get the information from the instruction manual based on the trial reports; (3) it reported the composition and the ratio of composition; or (4) it reported the patent proprietary CHM formula as the confidential formula that was applied in the trial.<br>"Partially or not reported" is considered if readers cannot know about the composition and dosage of the patent proprietary CHM formula in the trial.<br>"Not applicable" is considered if no patent proprietary CHM formula was applied in the trial.
	2. Illustration of the details of the formula, namely 1) the proprietary product name (i.e., brand name), 2) name of manufacturer, 3) lot number, 4) production date and expiry date, 5) name and percentage of added materials, and 6) whether any additional quality control measures were conducted	Q30. For trials with patent proprietary CHM formulas, whether the efficacy was reported in "Methods"?	"Full reported" is considered if (1) the efficacy of the patent proprietary CHM formula was reported; or (2) it reported the reference(s) as to the efficacy of the patent proprietary CHM formula.<br>"Partially or not reported" is considered if the efficacy of the patent proprietary of the CHM formula was Partially or not reported.<br>"Not applicable" is considered if no patent proprietary CHM formula was applied in the trial.

		Q31. For trials with patent proprietary CHM formulas, whether the safety or quality control was reported in "Methods"?	"Full reported" is considered if (1) the safety or quality control of the patent proprietary CHM formula was reported; or (2) readers can get the information from the instruction manual, references, or additional files based on the trial reports; (3) it reported the name of manufacturer and lot number of patent proprietary CHM formulas so readers can get the information from open materials.<br>"Partially or not reported" is considered if readers cannot know about the safety or quality control of the patent proprietary CHM formula in the trial.<br>"Not applicable" is considered if no patent proprietary CHM formula was applied in the trial.
		Q32. For trials with patent proprietary CHM formulas, whether the proprietary product name (i.e., brand name), name of the manufacturer, and lot number were reported in "Methods"?	"Full reported" is considered if the proprietary product name (i.e., brand name), name of manufacturer, and lot number were reported in Methods.<br>"Partially or not reported" is considered if the proprietary product name (i.e., brand name), name of manufacturer, or lot number were partially or not reported.<br>"Not applicable" is considered if no patent proprietary CHM formula was applied in the trial.
		Q33. For trials with patent proprietary CHM formulas, whether the production date and expiry date were reported in "Methods"?	"Full reported" is considered if 1) the production date and expiry date of the patent proprietary CHM formula were reported; or 2) readers can get the information from the instruction manual based on the trial reports.<br>"Partially or not reported" is considered if readers cannot get the production date and expiry date from the trial reports.<br>"Not applicable" is considered if no patent proprietary CHM formula was applied in the trial.
	3. Statement of whether the patent proprietary formula used in the trial is for a condition that is identical to the publicly available reference	Q34. For trials with patent proprietary CHM formulas, whether the patent proprietary formula used in the trial is for a condition that is identical to the publicly available reference was stated?	"Full reported" is considered if (1) it reported the patent proprietary formula used in the trial was for a condition that was identical to the publicly available reference(s) or the instruction manual; (2) it reported the trial aimed to explore the efficacy of patent proprietary CHM formula in treating a different disease or TCM pattern; or (3) it reported that the patent proprietary CHM formula targeted the disease or TCM pattern, and it given the references.

			"Partially or not reported" is considered if it did not report the patent proprietary CHM formula used in the trial was for a condition that was identical to the publicly available reference(s).<br>"Not applicable" is considered if no patent proprietary CHM formula was applied in the trial.
**5d. Control groups Placebo control**	1. Name and amount of each ingredient	Q35. For trials with placebo control, whether the name and amount of each ingredient of the placebo were reported in "Methods"?	"Full reported" is considered if (1) the name and amount of each ingredient of the placebo were reported; or (2) the name and ratio of each ingredient of the placebo were reported.<br>"Partially or not reported" is considered if the name or amount of each ingredient of the placebo was partially or not reported in Methods.<br>"Not applicable" is considered if no placebo control was conducted in the trial.
	2. Description of the similarity of placebo with the intervention (e.g., color, smell, taste, appearance, packaging)	Q36. For trials with placebo control, whether the similarity of placebo with the intervention (e.g., color, smell, taste, appearance, packaging) was reported in "Methods"?	"Full reported" is considered if (1) the similarity of placebo with the intervention (e.g., color, smell, taste, appearance, packaging) was reported; (2) it reported the specific information of both placebo and intervention, and readers can learn about the similarity of them.<br>"Partially or not reported" is considered if there is no information about the similarity of placebo with the intervention in Methods.<br>"Not applicable" is considered if no placebo control was conducted in the trial.
	3. Quality control and safety assessment, if any	Q37. For trials with placebo control, whether the quality control and safety assessment of the placebo were reported in "Methods"?	"Full reported" is considered if the quality control and safety assessment of the placebo were reported in Methods.<br>"Partially or not reported" is considered if there was no information about the quality control or safety assessment of the placebo in Methods.<br>"Not applicable" is considered if no placebo control was conducted in the trial.
	4. Administration route, regimen, and dosage	Q38. For trials with placebo control, whether the administration route, regimen, and dosage of the placebo were reported in "Methods"?	"Full reported" is considered if the administration route, regimen, and dosage of the placebo were all reported in Methods.<br>"Partially or not reported" is considered if the administration route, regimen, or dosage of the placebo were partially or not reported in Methods.<br>"Not applicable" is considered if no placebo control was conducted in the trial.

	5. Production information: where, when, how, and by whom the placebo was produced	Q39. For trials with placebo control, whether the production information of the placebo was reported, including where, when, how, and by whom the placebo was produced?	"Full reported" is considered if it reported the production information of the placebo, including where, when, how, and by whom the placebo was produced.<br>"Partially or not reported" is considered if the production information of the placebo was partially or not reported in Methods.<br>"Not applicable" is considered if no placebo control was conducted in the trial.
Outcomes	Illustration of outcome measures with Pattern in detail	Q40. Whether the outcome measures included TCM indicators in "Outcome"?	"Full reported" is considered if the outcome measures included TCM-related indicators, and it reported the name, measure methods (e.g. the formulas for calculating the incidence rate of TCM patterns/symptoms, scales to evaluate the TCM patterns/symptoms, or references to provide the methods to measure TCM-related outcomes), and measure point of the TCM indicators (specific date or indicated point).<br>"Partially or not reported" is considered if the name, measure methods, or measure point of the TCM indicators were partially or not reported in Methods.<br>"Not applicable" is considered if no TCM-related outcome was measured in the trial.
**Discussion** Generalizability	Discussion of how the formula works on different TCM Patterns or diseases	Q41. Whether any discussion of how the formula works on different TCM Patterns or diseases was reported in "Discussion"	"Full reported" is considered if it discussed how and when the CHM formula would work on different TCM Patterns or different diseases in the Discussion.<br>"Partially or not reported" is considered if it did not report how and when the CHM formula would work on different TCM Patterns or different diseases in Discussion.<br>"Not applicable" does not apply to this item.
Interpretation	Interpretation with TCM theory	Q42. Whether any interpretation with TCM theory was reported in "Discussion"?	"Full reported" is considered if the results were interpreted and discussed with the TCM theory in the Discussion.<br>"Not reported" is considered if there were no any discussion about the TCM theory in the Discussion.<br>"Not applicable" does not apply to this item.

*TCM: traditional Chinese medicine; CHM: Chinese herbal medicine
